# A Comprehensive Exploration of Caspase Detection Methods: From Classical Approaches to Cutting-Edge Innovations

**DOI:** 10.3390/ijms25105460

**Published:** 2024-05-17

**Authors:** Mahmoud Zhra, Rani J. Qasem, Fai Aldossari, Rimah Saleem, Ahmad Aljada

**Affiliations:** 1 Department of Biochemistry and Molecular Medicine, College of Medicine, Alfaisal University, Riyadh 11533, Saudi Arabia; 2 Department of Pharmacology and Pharmacy Practice, College of Pharmacy, Middle East University, Amman 11831, Jordan; 3 Zoology Department, College of Science, King Saud University, Riyadh 12372, Saudi Arabia

**Keywords:** apoptosis, caspases, high-content, high-throughput screening, mass spectrometry, antibody-based assays, florescence based detection methods, in vivo imaging techniques, real-time monitoring methods

## Abstract

The activation of caspases is a crucial event and an indicator of programmed cell death, also known as apoptosis. These enzymes play a central role in cancer biology and are considered one promising target for current and future advancements in therapeutic interventions. Traditional methods of measuring caspase activity such as antibody-based methods provide fundamental insights into their biological functions, and are considered essential tools in the fields of cell and cancer biology, pharmacology and toxicology, and drug discovery. However, traditional methods, though extensively used, are now recognized as having various shortcomings. In addition, these methods fall short of providing solutions to and matching the needs of the rapid and expansive progress achieved in studying caspases. For these reasons, there has been a continuous improvement in detection methods for caspases and the network of pathways involved in their activation and downstream signaling. Over the past decade, newer methods based on cutting-edge state-of-the-art technologies have been introduced to the biomedical community. These methods enable both the temporal and spatial monitoring of the activity of caspases and their downstream substrates, and with enhanced accuracy and precision. These include fluorescent-labeled inhibitors (FLIs) for live imaging, single-cell live imaging, fluorescence resonance energy transfer (FRET) sensors, and activatable multifunctional probes for in vivo imaging. Recently, the recruitment of mass spectrometry (MS) techniques in the investigation of these enzymes expanded the repertoire of tools available for the identification and quantification of caspase substrates, cleavage products, and post-translational modifications in addition to unveiling the complex regulatory networks implicated. Collectively, these methods are enabling researchers to unravel much of the complex cellular processes involved in apoptosis, and are helping generate a clearer and comprehensive understanding of caspase-mediated proteolysis during apoptosis. Herein, we provide a comprehensive review of various assays and detection methods as they have evolved over the years, so to encourage further exploration of these enzymes, which should have direct implications for the advancement of therapeutics for cancer and other diseases.

## 1. Caspases: Caspases Play a Crucial Role in Regulating Cellular Death and Coordinating Complex Communication Pathways in Both Normal Physiological Processes and Pathological Conditions

Caspases, a group of cysteine-dependent proteases, have emerged as crucial regulators of programmed cell death, commonly referred to as apoptosis [1,2]. Extensive research spanning decades on the cellular biology of these enzymes has demonstrated their crucial role in maintaining overall cellular health. The regulation of caspase activation is carefully managed, and any disturbances in this process can result in the onset and advancement of a range of diseases, including cancer and neurodegeneration [3]. Research on caspase inhibitors and activators demonstrates encouraging results for potential clinical use, indicating that targeting these molecular regulators could become an important therapeutic approach for specific illnesses [4].

**Figure 1:**
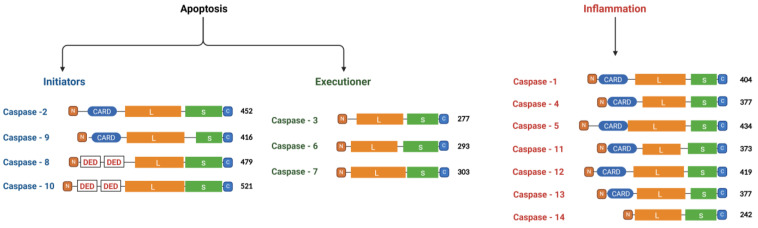
Classification of caspases in mammals based on their domain structures and functions. The apoptotic initiator caspases, namely caspase-2, -8, -9, and -10, are responsible for initiating the apoptotic process. On the other hand, the apoptotic executioner caspases, including caspase-3, -6, and -7, execute apoptosis. Inflammatory caspases, such as caspase 1, 4, 5, 11, 12, 13, and 14, are involved in inflammatory responses. The domain compositions of caspases are diverse and involve CARD and DED domains. These domains play crucial roles in protein–protein interactions and signaling pathways. Caspases exhibit a modular structure consisting of a large (L) and a small (S) subunit. Some caspases also feature short (S*) and long (L*) subunit variants, contributing to their functional diversity and regulation.

Caspases usually cleave peptide bonds following aspartate residues [4,5]. The specificity of this cleavage is crucial during apoptosis and inflammation [6]. In addition, the proteolytic activity of these enzymes involves the cleavage of vital cellular substrates and is sufficient to initiate the morphological changes associated with apoptosis [6,7]. Furthermore, caspases have also been shown to have a crucial function in merging apoptotic and inflammatory pathways, finely tuning cell death and innate immune responses [7]. Because apoptosis is an evolutionarily conserved process, caspases have been found in many species, ranging from worms to humans [8]. The human caspase family was initially identified through the discovery of caspase-1 and comprises 14 members [9]. These members are categorized into three distinct groups based on sequence homology, substrate consensus sequence, and their position in the apoptotic cascade. The first group is the initiator caspases (caspase 2, 8, 9, and 10), which are responsible for initiating apoptotic pathways. The second group is the executioner caspases (caspase 3, 6, and 7), which play a role in carrying out the apoptotic program. The third group is the inflammatory caspases (caspase 1, 4, 5, 11, 12, 13, and 14), which are involved in inflammatory responses [3,10,11]. Each of these caspases exhibits unique substrate specificities [12]. Like many proteases, these enzymes are initially synthesized as inactive zymogens and undergo a series of signaling events that include an internal cleavage at specific Aspartic acid (Asp) residues to attain activation [3,13]. Structurally, the enzymes are composed of a prodomain and a large (p20) and small (p10) catalytic subunits (Figure 1) [6,14]. Furthermore, every caspase contains a preserved pentapeptide active-site motif with the sequence QACXG (where X represents either R, Q, or G) located in the large catalytic subunit that is crucial for its proteolytic function [7]. The caspase recruitment domain (CARD) and death effector domain (DED) are small interaction domains present in caspases and their target proteins, playing a vital role in caspase activation during apoptosis [15]. A key component in the initiation and regulation of downstream signaling in apoptosis and inflammation is the apoptosome, a large molecular complex formed by the interaction of the apoptotic protease-activating factor-1 (APAF-1) with caspase-9 through their respective CARD domains. This interaction exemplifies a single CARD-containing target protein of caspases within the apoptotic signaling cascade [16,17,18,19]. The DED domain is present in procaspases 8 and 10, which are inactive, as well as in target adaptor proteins like the FAS-associating death domain-containing protein (FADD). Its role in apoptosis involves crucial functions such as caspase recruitment, regulation, and activation [20,21,22]. Of the caspases, caspase-11 has been shown to play a critical role in the innate immune response against bacterial infections, while Caspase-13 serves as the bovine counterpart to caspase-4 [23]. Caspase-12 specifically operates within the endoplasmic reticulum (ER) and is activated by ER stress, often triggered by disruptions in ER calcium balance [24]. However, it is worth mentioning that ER stress-induced apoptosis can also occur through a mechanism that does not solely rely on caspase-12 but involves the apoptosome [24]. On the other hand, caspase-14 is a developmentally regulated protease, highly expressed in embryonic tissues and implicated in apoptosis [25].

**Figure 2:**
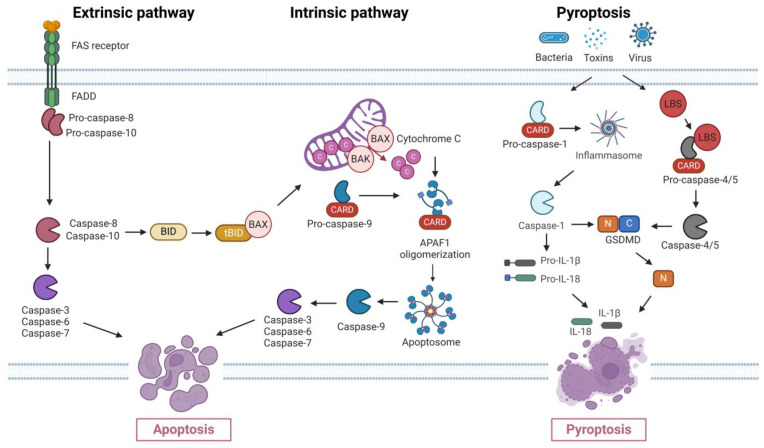
The molecular mechanisms of cell death controlled by Caspase-3, encompassing both classical and non-classical apoptosis pathways. This elucidates the diverse cellular responses to internal and external signals. This comprehensive understanding includes key apoptotic players such as BID, BAX, APAF1, and BAK, revealing their roles in the classical pathway. BID is a crucial link between extrinsic and intrinsic signals, while BAX and BAK contribute to mitochondrial permeabilization, releasing cytochrome C. Additionally, the figure illustrates the caspases, notably Caspase-1 and Caspase-4/5, in pyroptosis, exemplified by their cleavage of Gasdermin-D. This process releases Gasdermin-D N-terminal (GSDMD-N), triggering the release of pro-inflammatory cytokines interleukin-1β (IL-1β) and interleukin-18 (IL-18) and inducing pyroptosis. The intricate interplay between caspases and their effectors is a pivotal switch determining cell fate, whether it undergoes apoptosis or transitions into pyroptosis.

Caspase activation occurs through two primary pathways: the extrinsic and intrinsic pathways (Figure 2) [26,27]. The intrinsic pathway is centered around forming a complex between APAF-1 and cytochrome c, which regulates the activation of procaspase-9 and ultimately leads to the activation of downstream caspases [28]. Conversely, the extrinsic pathway is activated by an external signal that interacts with surface death receptors like Fas and tumor necrosis factor (TNF) receptors. This activation leads to the initiation of caspase-8, which can subsequently activate other caspases or induce the release of cytochrome c from the mitochondria into the cytoplasm [29]. In the intrinsic pathway, a well-defined hierarchy exists, wherein caspase-9 undertakes the processing and activation of effector caspases, including caspase-3 and -7. These activated effector caspases then proceed to process other caspases in a sequential manner [28]. The complex interaction of caspases in both pathways is impacted by a variety of proteins, such as a group of proteins referred to as inhibitors of apoptosis (IAP) [26,29].

In the regulation of cell death, caspase-3 is identified as a key protease responsible for carrying out the final stages of apoptosis [30]. Caspase-3 plays a significant role not only in the execution of apoptosis but also in the intricate interplay between caspases, autophagy, and apoptosis. Acting as a central molecular switch, this enzyme enables the dynamic communication between these different cellular pathways [31]. On the other hand, caspase-9 has been recognized for its non-apoptotic functions in the modulation of autophagy, suggesting that apoptosis and autophagy are intertwined systems that intersect during the process of cellular death [32,33]. Disruption of the complex interaction among these pathways and their components has the potential to cause significant pathophysiological ramifications [34]. Hence, it is clear that a deeper exploration into the molecular mechanisms that govern caspase activation and regulation in apoptosis, as well as their interactions within the cell and communication with other signaling pathways, results in a more comprehension of apoptosis as a biological process. This enhanced understanding also opens up numerous possibilities for therapeutic interventions, particularly in cancer treatment [35].

Caspases also have a critical role in the initiation of pyroptosis, a specific form of programmed cell death characterized by cellular inflammation and eventual lysis [36]. Pyroptosis is triggered by inflammatory caspases, specifically caspase-1/-4/-5 (human) and -11 (mice) [37]. The process involves the formation of pores in the plasma membrane that cause the osmotic lysis of cells, DNA damage, and activation of ADP-ribose polymerase [38,39]. A key event in the induction of pyroptosis is the cleavage of Gasdermin-D by inflammatory caspases [37]. This process serves as an innate immune response mechanism against intracellular bacterial infections, eliminating bacteria from the host [40,41]. Furthermore, the correlation between pyroptosis and the death of cancer cells has been confirmed, indicating potential implications in the field of cancer therapy. The intricate interplay between caspases and pyroptosis highlights the diverse roles these enzymes play in regulating cellular functions in response to various external triggers. Again, comprehending these complex mechanisms enhances the understanding of fundamental biological processes and provides opportunities for targeted interventions in situations where disruptions in cell death pathways, including pyroptosis, play a crucial role. 

The role of caspases in suppressing and promoting neoplastic transformation has garnered particular attention in recent years [42]. Researchers have been actively exploring the potential of targeting caspases for cancer treatment to reactivate apoptotic signaling and overcome therapeutic resistance [43]. Therefore, there is growing interest in unraveling the complex relationships between caspases and key apoptotic mediators [44]. The ongoing investigation into caspase-related strategies has yielded encouraging results, offering great promise for advancing precision medicine and improving treatment outcomes for specific types of cancers. In order to unravel the intricate mechanisms involved in regulating these enzymes and identify potential targets for tailored therapeutic interventions, the availability of high-throughput screening methods to assess caspase activities, which can be automated, is crucial. Such methods can provide novel insights into caspase function and aid in the development of more effective treatment approaches.

## 2. Classical Approaches to Detecting Caspase Activity

### 2.1. Early Days: Pioneering the Path (1990s–Early 2000s)

Early efforts depended on time-consuming and indirect approaches such as morphological analysis and DNA fragmentation assays. Nevertheless, the demand for a targeted and accurate tool drove the advancement of antibody-based methods. The initial phase involved Western blotting, which provided data on protein levels but did not offer accurate quantification of activity [45]. The antibodies’ specificity, user-friendly nature, heightened sensitivity, and ability to semi-quantify caspase proteins in cell lysates and tissues gave them widespread recognition by many prominent researchers. This justifies why antibody-based assays are considered indispensable tools for caspase research. Perhaps this is evident in the work described by McStay and Green, who used various antibodies to specifically identify activated executioner caspases [45]. The application of antibodies targeting cleaved caspase-3 represented a significant advancement in identifying and quantifying apoptotic cells, especially in the immunohistochemical (IHC) evaluation of well-preserved paraffin-embedded tissue sections [46,47]. The IHC emerged as a technique that enables the visualization of caspase activation within tissues, providing significant spatial context [46].

### 2.2. Refinement and Innovation (Mid-2000s–Present)

The sensitivity and quantification capabilities of immunoassays have been greatly enhanced by the development of more advanced antibody-based methods, such as enzyme-linked immunosorbent assay (ELISA) and Luminex [45]. In addition, the antibodies have been effectively used in detecting apoptosis using flow cytometry-based techniques [48], which allow the detection of caspase activity in individual cells, providing valuable insights into the heterogeneity of cell populations [49]. The use of intracellular binding molecules, such as intrabodies, has also expanded live cell imaging capabilities, enabling real-time cell-based diagnostics [50]. Furthermore, developing small molecule active site-directed tools has opened up new avenues for studying human caspases [51]. These advancements have paved the way for the development and validation of flow cytometry methods for clinical pharmacodynamic biomarkers, thereby enhancing the potential of these tools in biomedical research [52].

Progress in enzyme assays has significantly increased sensitivity and the capacity to perform high-throughput screening. Luminescent substrates, especially those utilized in caspase activity assays, have notably improved sensitivity and allowed for measurements with just one addition [53]. High-throughput enzyme assays have been developed using labeled substrates and indirect sensor systems, with recent advancements in labels and chromophores [54]. Nanomaterial-based optical biosensing has been utilized to detect caspase-3 activity, which is essential in apoptosis [55]. Bioluminescent assays are also used for high-throughput screening, providing sensitivity and reliability [56]. Furthermore, fluorescence quenching-based assays have been developed for hydrolyzing enzymes, such as caspases [57]. Recombinant DNA technology has been employed to enhance enzyme assays [58]. The creation of caspase-specific inhibitors has improved assay specificity and simplified the identification of distinct caspase family members involved in apoptosis [59]. Lastly, a high-throughput screen platform has been established to discover the natural substrate range of caspases [60].

However, antibody-based assays have several limitations, emphasizing the need for continuous improvement in detection methods. These include their inability to fully capture the details of the steps involved in apoptosis, which is needed to develop a global understanding of the entire process. In addition, the lack of specificity in many of the commonly used caspase antibodies is a frequently encountered issue that can complicate the interpretation of experimental data and requires extensive, time-consuming validation [46,61,62]. Due to the continuous expansion of investigations on caspases and the discovery of new targets and regulation mechanisms, many of which were previously unknown, the experimental utility of antibody-based assays as tools of scientific investigation is falling behind and becoming increasingly limited. Therefore, there is a growing demand to improve existing detection techniques and introduce new approaches that can overcome the limitations of conventional antibody-based assays. These new methods aim to offer a more thorough, detailed, and precise insight into caspase activities across various cellular environments [63,64,65].

## 3. Evolution and Revolution in Caspase Detection

Detection methods for caspases have evolved significantly, transitioning from traditional antibody-based techniques to more advanced, sensitive, and innovative approaches. Below is a description of these newer methods.

### 3.1. Fluorescent Probes for Caspases

**Figure 3:**
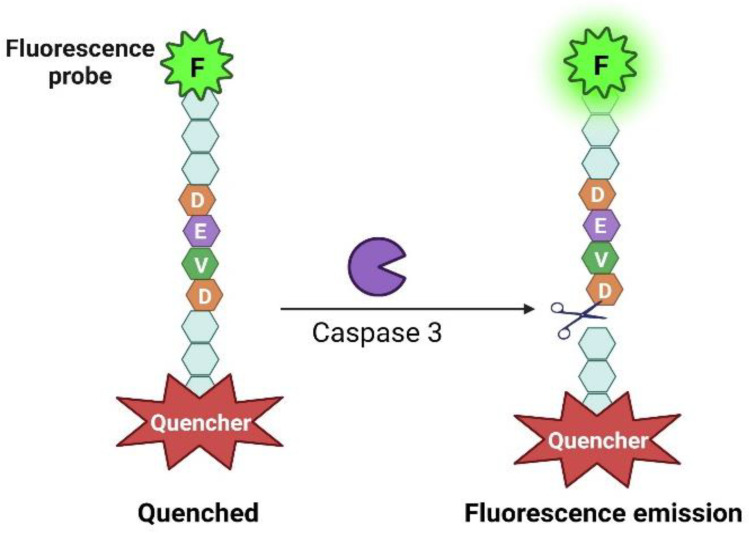
The fluorescence probe-based assays depicted in the illustrated schematic diagram have been developed to enable the sensitive detection of caspase-3 activity. This approach involves the activation of caspase-3, which leads to the cleavage of the substrate at a specific location. As a result, the fluorophore and quencher molecules become separated, leading to fluorescence emission. This emitted fluorescence is a reliable and easily distinguishable indicator, allowing for visual and quantitative analysis of active caspase-3.

The introduction of caspase-sensitive fluorescent probes in 1982 represented a breakthrough in detection methods for basal and apoptosis-related caspase activities (Figure 3) [66]. Initially, researchers utilized a molecular probe consisting of Cyan Aequorea fluorescent proteins (CFP) and Yellow fluorescent protein (YFP) fluorophores joined together through a peptide linker that harbors two caspase cleavage sites. The proximity of the two fluorophores allows for fluorescence resonance energy transfer (FRET) upon excitation, causing intense emission. Following the activation of caspases and cleavage of the peptide linker, the emission faded as the CFP and YFP moieties parted ways. This probe allowed for real-time tracking of caspase activity within living cells by flow cytometry [66]. Another research group created a series of fluorescently labeled, activity-based probes that targeted different caspases. These probes formed permanent covalent bonds with the active target caspase. They allowed the non-invasive in vivo imaging of the kinetics of apoptosis to facilitate the monitoring of disease progression and chemotherapeutic response [67]. Another activity-based probe specific for caspase-1 was developed using reverse design principles, based on the structure of chemically optimized protease inhibitors. These inhibitors were transformed into selective activity-based bioluminescent probes that produced amino-luciferin upon cleavage by caspase [68]. Advancements in probe design also involved the introduction of cell-permeable ratiometric fluorescence imaging probes, which enable the measurement of biological parameters by detecting changes in the ratio of emitted fluorescence at two different wavelengths. These probes internalize into the cell and display ratiometric changes in fluorescence following cleavage, allowing the quantification of caspase activity and real-time monitoring of apoptosis without cellular permeabilization [69]. These techniques have enhanced the ability to investigate and comprehend caspase activity.

**Figure 4:**
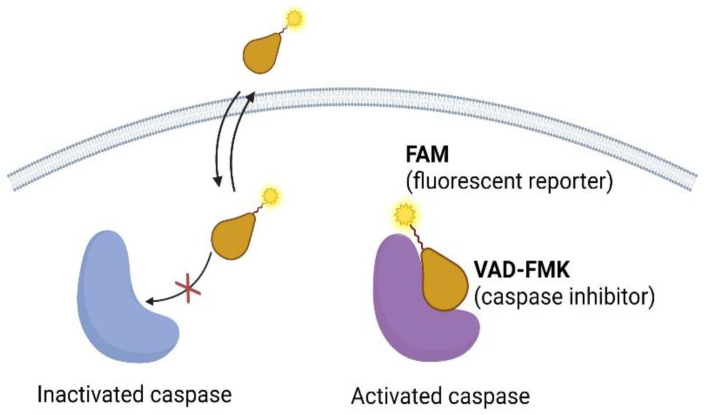
The diagram illustrates using FLICAs to selectively label live cells with activated caspases. The main focus is on the polycaspase inhibitor FAM-VAD-FMK, an example of a FLICA. An essential step on the left side of the diagram is highlighted, which involves washing the cells to remove any unbound FLICAs. This washing step is crucial to ensure that only the FLICAs specifically bound to activated caspases remain on the cells, allowing for accurate visualization and detection. To the right side of the diagram, the FLICAs, including FAM-VAD-FMK, are shown to attach to activated caspases in the live cells. This specific binding mechanism enables the visualization and detection of cells with activated caspases through fluorescence. The fluorochrome label attached to the FLICAs emits fluorescence when excited by a particular wavelength of light, making it possible to identify and track cells with activated caspases. Using FLICAs to selectively label live cells with activated caspases is beneficial for monitoring caspase activation in real time. By observing the fluorescence emitted by the FLICAs, researchers can track the activation of caspases and gain insights into apoptotic processes.

On another front, the development and application of fluorochrome-labeled inhibitors of caspases (FLICAs) for apoptosis detection was a collaborative effort that involved contributions from multiple researchers and laboratories (Figure 4). The principle of FLICAs is based on the binding of carboxyfluorescein (FAM)- or fluorescein (FITC)-tagged peptide-fluoromethyl ketone (FMK) to the active substrate binding site of esterases and proteases [70,71]. FLICAs contain different peptide moieties that can bind to activated caspases, such as -2, -3, -6, -1, -8, -9, and -10 [72].

In this instance, Jayaraman and Lee reported two notable investigations on FLICAs for caspase detection [73]. Jayaraman’s research primarily focused on flow cytometry-based methods, while Lee demonstrated the applications of FLICAs for both in vitro and in vivo apoptosis detection. These FLICAs bind irreversibly to active caspases and are associated with a noticeable change in their fluorescence properties. As a result, caspase activation can be visualized with exceptional temporal resolution at the individual cell level [74]. However, caution has been raised on the specificity of FLICAs in detecting caspase activation by Pożarowski [75], Berger [76], and Darżynkiewicz [77]. Despite this concern, Grabarek [78] and Darzynkiewicz [79] successfully demonstrated the rapid and accurate detection of apoptotic cells with FLICAs, highlighting their utility in caspase studies. The utilization of FLICAs has been expanded to encompass high-content screening for identifying regulators in the apoptotic machinery [80]. Moreover, FLICAs have been employed for investigating the intracellular localization of caspases to help establish a correlation between their activation, cell cycle position, and other cellular attributes that otherwise would require cellular permeabilization for measurement [81]. The continuous advancement in FLICAs highlights their utility and adaptability for detecting caspase activity in laboratory and living organisms. Although the use of FLICAs is an effective method for detecting apoptosis without causing cell toxicity, it has been reported that the FMK group within FLICAs can interact with intracellular protein thiol groups, reducing its specificity [75,77,82].

### 3.2. Evolution of Laser Scanning Cytometry (LSC)

The principle of LSC relies on the fluorescence intensity or loss of laser light (absorbance) of localized molecular targets measured within the nuclear and cytoplasmic structures of cells while maintaining the morphological features of the examined tissue. It combines the cell imaging capability of flow cytometry with several distinct advantages. It also helps overcome many of the limitations of flow cytometry. Telford et al. were pioneers in using LSC to detect apoptosis, especially in detecting early apoptotic cells. This technique has since been widely adopted for the precise and quantitative analysis of early apoptotic cells [83]. LSC can measure several parameters, such as integrated fluorescence intensity, maximal pixel, integration area, the perimeter of the integration contour, circularity, and fluorescence intensity, which have been explained in detail by Pozarowski et al. [84].

In addition, the LSC can identify low-level, localized caspase expression, which is a crucial indicator of apoptosis; LSC also facilitates the measurement of the changes in cell morphology, plasma membrane composition, and mitochondrial transmembrane potential. These factors are closely associated with apoptosis [81,85,86]. Importantly, LSC is a cost-effective and reliable method for studying caspase-mediated apoptosis with standard caspase substrates and inhibitors [87], and the integration of FLICAs in LSC offers an additional enhancement to the sensitivity and specificity of the method in detecting caspase activity [81]. The ability of LSC to separately determine nuclear and cytoplasmic components, along with assessing the localization and expression of specific markers, is noteworthy [84].

### 3.3. Breakthroughs in Homogeneous Caspase Activity Detection

Homogeneous assays have seen significant progress in detecting caspases, particularly caspases 3 and 7, in the last twenty years. These assays combine all reaction components into a single phase, eliminating the need for separation steps like washing or centrifugation. Various vendors now offer a wide range of these assays with improved sensitivity, many of which are optimized for high-throughput screening and seamless integration into automated processes. While the underlying principle of these assays remains the same—relying on light emission in response to caspase activity—the specific molecular mechanisms leading to this emission can differ between assays from different vendors, giving each assay its unique characteristics.

A critical development in homogeneous bioluminescent protease assays was the introduction of the novel caspase substrate, Z-DEVD-conjugated aminoluciferin, which is used along with a molecularly stabilized luciferase in a simplified single-step assay format. This combination enabled simultaneous protease cleavage of the substrate and luciferase-mediated oxidation of aminoluciferin such that equilibrium was rapidly achieved and consistent luminescence was maintained for hours. Compared to conventional fluorescent assays, this innovative design offered notable benefits, including faster outcomes and enhanced sensitivity [88].

**Figure 5:**
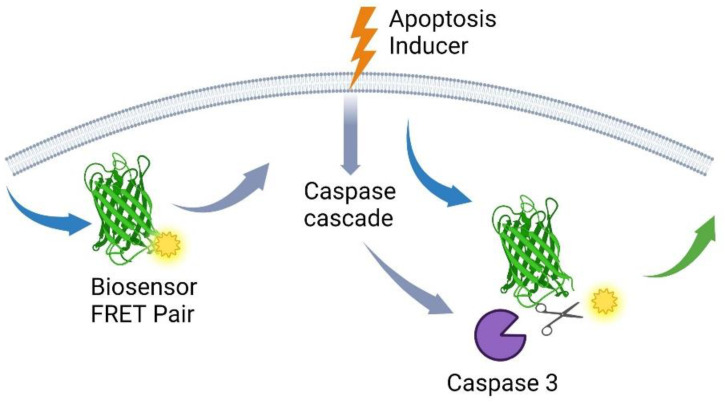
A specialized bioprobe is designed with a caspase-3 recognition sequence linked to a FRET pair. This bioprobe is then introduced into the cellular environment. When apoptosis is initiated, the activated caspase-3 specifically cleaves the bioprobe, causing the separation of the fluorescent molecules that make up the FRET pair. This crucial cleavage event is carefully detected by measuring the fluorescence emission in both the donor and acceptor channels. The illustration also highlights the importance of the lipid bilayer, emphasizing its significant role in creating a physiologically relevant cellular environment for the apoptosis process.

Another two innovative homogenous assays of improved sensitivity that are worth mentioning and have been used in detecting the activities of caspase-3 and 7 are the Lanthanide Chelate Excitation (LANCE) and homogeneous time-resolved FRET assays. The LANCE method utilizes a luminescent europium chelate-labeled cleavable peptide combined with a quencher. The peptide cleavage liberates the europium chelate from the quencher, allowing it to resume its fluorescence properties. This design ensures enhanced sensitivity and improved specificity in detecting caspase-3 activity [89]. On the other hand, the homogeneous time-resolved FRET assay uses a double-tagged caspase substrate carrying an energy donor and acceptor to help resolve fluorescence emitted through energy transfer with time (Figure 5) [90]. However, the assay can cause a cross-talk between the two FRET probes. This issue can be resolved by a novel ratiometric protease sensor developed for caspase-3 using a single enhanced green fluorescent protein (EGFP) molecule. This innovative approach takes advantage of EGFP’s remarkable resistance to proteases and strategically positions a caspase-3-specific cleavable linker at a sensitive loop location (Glu172) near the EGFP chromophore. The sensor could detect caspase-3 activation or inhibition in cell-based systems and identify the early stages of apoptosis with no interferences from the background expression level [91].

Regarding the prospect of improving the sensitivity and accuracy of these assays, several approaches have been developed over time to eliminate background autofluorescence originating from the sample. One method uses an upconverting phosphor (UCP) donor that emits visible light upon excitation at near-infrared and a conventional fluorophore acceptor on the caspase-3-specific substrate peptide. A quencher molecule attached to the peptide attenuates the acceptor’s emission in the intact peptide through intramolecular energy transfer. However, under non-inhibitory conditions, peptide cleavage separates the fluorophore from the quencher, thereby restoring the emission [92]. Using the UCPs and quencher in this assay reduced autofluorescence and background radiation, improving the assay’s signal-to-background ratios. These assays offer high sensitivity and rapid means for detecting caspase 3 activity in high-throughput screening platforms for drug discovery.

Besides the aforementioned methods for homogeneous detection of caspase-3 activity, there have been endeavors to create label-free sensors for homogeneous detection. An instance of an innovative label-free and blocker-free strategy is the photoelectrochemical (PEC) sensor, which employs bifunctional CC-DEVD-peptide modified nitrogen-doped porous carbon-ZnO nanopolyhedra/CdS hybrids. It has been documented that this sensor enhances caspase detection limits [93]. In addition, attempts to integrate nanomaterials in the design of these sensors helped improve their specificity, precision, and sensitivity [94]. For example, gold nanoparticle-functionalized mesoporous materials have been used to design ultrasensitive electrochemical biosensors to detect caspase-3 activation during hematopoietic stem cell differentiation [95]. The utility of these nanomaterial-modified sensor platforms is most illustrated in the detection of early biomarkers of cancer, which can help diagnose patients during the early stages of the disease [96]. Organic electrochemical transistor biosensors have also been utilized for the high-sensitivity detection of caspase-3 activity, with a detection limit down to 0.1 pM [97].

A range of protease detection methods that do not require labeling have also emerged, indicating a significant shift in the experimental approach for analyzing protease activity. One notable advancement in this field is developing a label-free surface plasmon resonance (SPR) method to detect the caspase-3 activity [98]. This SPR platform is highly versatile and seamlessly integrated into various experimental setups. It is also adaptable for detecting caspase activity in homogeneous and heterogeneous environments [99,100]. The flexibility of SPR technology offered valuable insights into the dynamic landscape of caspase reactions and the biomolecular interactions involved, especially in a high-throughput format [101,102]. This emphasizes the broader utility of SPR technology in offering real-time and multiparametric insights into the molecular interactions implicated in cellular processes. SPR Microscopy is also a powerful tool for studying molecular interactions, but it differs from traditional SPR in its applications and capabilities. Traditional SPR measures changes in refractive index and is used for real-time monitoring of binding kinetics and analyte concentrations [103]. In contrast, SPR Microscopy offers high spatial resolution and the ability to visualize molecular interactions, making it useful for studying complex biological samples [104]. Two independent studies have demonstrated the potential of SPR imaging for monitoring caspase reactions [98,101]. One study achieved an impressive detection limit of 1 pg mL(-1) for caspase-3 [105]. The utility of this approach is evident in another study that employed an SPR imaging protein chip system to monitor the caspase-3 activation [98]. In addition, Zhang [106] and Li [107] explored using SPR biosensors and a nonconjugated gold nanoparticle-quantum dot pair for detecting mismatched caspase-3 DNA oligonucleotides and caspase-3 activities, respectively.

The convergence of these label-free techniques, supported by the SPR platform, represents a significant advancement in protease detection and has deepened the understanding of caspase-related phenomena. These methods align with the broader trend of developing label-free methods for protease detection, which encompass other innovative detection approaches such as peptide-templated gold nanoclusters, peptide-based electrochemical biosensors, bioluminescent nanosensors, fluorescent-conjugated polymer super quenching, protein-protected Au clusters, and microplate-based detection of biomolecular interactions. Renowned for their high sensitivity, specificity, and rapid detection capabilities, these methods are invaluable in clinical diagnostics and therapeutics.

### 3.4. In Situ Assessment of Caspase Activity

The development of methodologies for in situ assessment of caspase activity has revolutionized the comprehension of this vital biological process. Numerous state-of-the-art techniques have been devised to aid in the visualization and quantification of caspase activity in living organisms.

#### 3.4.1. Multifunctional Probes for In Vivo Imaging

The development of activatable multifunctional probes for in vivo imaging of caspase-3 represents a significant advancement in detecting and monitoring caspase activity, particularly in assessing the early effects of therapeutics on tumors [108,109]. When formulated into nanoparticles, these probes have effectively overcome the challenge of penetrating cells, allowing for precisely labeling cells in response to apoptotic signals [108]. In addition, the use of ratiometric fluorescence imaging probes enables the real-time quantification of caspase-3 activity, which can offer valuable insights into the stages of apoptosis in living cells [69]. The innovative application of bio-orthogonal cyclization-mediated in situ self-assembly of small molecule probes has further enabled the in vivo imaging of caspase activity, particularly in human tumor xenograft mouse models undergoing chemotherapy [110]. The optimization of activity-based probes has played a pivotal role in advancing the comprehension of caspase-6 activation. It has aided in uncovering distinctive facets of the mechanisms involved in its activation and biological functions [111]. Lastly, a combined approach of protein and probe engineering has been utilized for the selective inhibition and labeling of caspases, which enables the imaging of individual caspases within the cells [112].

#### 3.4.2. Single-Molecule Spectroscopy Insights

The emergence of single-molecule spectroscopy has revolutionized the field of caspase research, enabling thorough examination of individual caspase molecules and the detection of subtle variations in their activity [113]. This breakthrough has stimulated the development of activity-based probes specifically designed for in vivo caspase detection, facilitating the visualization and quantification of caspase activity at the single-molecule level [69]. The profound impact of single-molecule spectroscopy is evident in its contribution to the understanding of caspase molecules, driving the refinement of activity-based probes to enhance selectivity and sensitivity in diverse biological contexts [51,114]. The advancement of small molecule active site-directed tools has furthered the exploration of caspase activity and specificity [115]. Notable discoveries include the identification of early caspase activation intermediates, such as a full-length caspase-7 intermediate, and developing highly selective inhibitors and active site probes [115]. These advancements have significantly deepened the comprehension of caspase molecules and helped unravel their roles in various biological processes.

#### 3.4.3. Real-Time Monitoring Techniques

Various advanced real-time monitoring techniques have been established to evaluate caspase activity in living cells. These encompass a range of methods; the ones addressed here are self-assembling nanofiber probes [116], fluorescent light-up probes with aggregation-induced emission characteristics [117], novel multicolor gold-selenium bonding fluorescent nanoprobes [118], and genetically encoded caspase sensors for live imaging [119]. Self-assembling nanofiber probes, named Nap-GFFpYDEVD-AFC and Nap-GFFpYIETD-AFC, are characterized by their unique fluorescence ‘turn-on’ properties. These probes have been specifically designed to enable the real-time tracking of Caspase-3/8 activity within living cells [117]. On the other hand, the probes featuring aggregation-induced emission properties were illuminated upon exposure to fluorescent light and enabled the early identification of diseases associated with apoptosis and facilitated drug monitoring. This probe can permeate cells and comprises three components: a hydrophilic peptide modified with DEVD, a hydrophobic tetraphenylethene, and an aggregation-induced emission (AIE) fluorogen. The probe exhibits a strong fluorescence signal in apoptotic cells, especially those containing active caspase-3/7 protein, thereby enabling a precise imaging [117]. The novel fluorescent nanoprobe GNP-Se-Casp, developed by Liu et al., utilized Au-Se bonding to observe caspase-3, 8, and 9 activities during cell apoptosis. By employing real-time fluorescence imaging on staurosporine-induced apoptotic cells, the nanoprobe showed sequential activation of fluorescence signals for caspase-8 and caspase-9, followed by caspase-3 fluorescence. Therefore, these GNP-Se-Casp probes can be highly effective in the real-time monitoring of the caspase cascade activation during tumor cell apoptosis [118]. The last approach, tailored for live imaging, is the use of genetically engineered caspase sensors. These sensors utilize fluorescence indicators to monitor caspase-3-like enzyme activity in multicellular environments. The cleavage by these enzymes indicates the transition from being non-fluorescent to fluorescent. In cultured cells, the healthy ones remained non-fluorescent. At the same time, apoptotic stimuli induced a notable fluorescence increase in the dead cells, offering insights into the impact of the cancer cell environment on drug sensitivity [119]. These techniques and similar ones have demonstrated their effectiveness in monitoring the activation of the caspase cascade and hold great promise for applications in cancer research, clinical detection, and the discovery of drugs targeting apoptosis as a cellular process.

#### 3.4.4. High-Content Screening of Caspase Activity

High-content screening assays targeting caspase activity provide a comprehensive understanding of apoptotic cells [80]. These assays allow for the simultaneous evaluation of various parameters, including caspase-specific activities [80,120], which is particularly valuable for drug screening and defining pathways [120]. Several methods have been developed for this purpose, including the cleavage of synthetic substrates and flow cytometry [121,122,123]. The successful integration of high-throughput screening for caspase activity with anti-apoptosis genes has created robust cell lines to produce biotherapeutics [124]. Furthermore, preclinical and clinical settings have demonstrated the noninvasive optical imaging of apoptosis using caspase-targeted probes [67]. Microfluidic devices, known for their precision in manipulating and analyzing cells, are a powerful platform for high-throughput screening of caspase activity in living cells [125,126]. These devices also enable the exploration of interactions between different cell types and the cultivation of tissue specimens under controlled microenvironments [127]. Their application extends to high-throughput screening and the development of biomaterials, replicating natural microenvironments and producing biomaterials with controlled properties [125]. The incorporation of electrochemical methods in these devices enhances sensitivity and enables label-free detection capabilities [125]. The miniaturization and parallelization of biological and chemical assays in microfluidic devices significantly increase throughput while reducing costs [128]. These characteristics make microfluidic devices promising tools for high-throughput screening and drug discovery. Overall, these studies highlight the essential role of high-content screening assays in understanding apoptotic cells.

#### 3.4.5. Mass Spectrometry (MS) in Caspase Activity Detection

The emergence of MS has brought about a groundbreaking revolution in identifying, detecting, and quantifying protein expression levels and evaluating posttranslational modifications and regulation of enzyme activities [94,129,130,131,132]. This technology has proven to be unparalleled in its sensitivity, specificity, resolution, and high-throughput screening, as well as its adaptability to achieving various experimental endpoints and platforms, allowing it to become a leading tool in protease research, including caspases. The applications of MS in caspase research can be classified into four different domains, showcasing its multifaceted contributions:

##### Detection of Caspase Enzyme Activity by MS

Several label-free MS methods have been reported for the detection of caspase activation. These methods offered several advantages regarding sensitivity, specificity, and convenience. One such method abbreviated SAMDI-MS, which stands for “self-assembled monolayers for matrix-assisted laser desorption ionization time-of-flight mass spectrometry”, used self-assembled monolayers of alkanethiolates on gold with immobilized peptide substrates for either caspase -3 or -8. To measure the endogenous activities for these caspases, the cellular extract is spotted onto the monolayers, then incubated and rinsed. The extent of substrate cleavage by caspases is then quantified by mass spectrometry. This method is as sensitive as the fluorogenic assay. In addition, the method offered improved specificity for each of the measured caspases since using longer peptide substrates provided better resolution between the activities of the two enzymes [129]. Another study has reported a similar method for assessing caspase activity using MS. In this method, the N-terminal cleavage product of an unlabeled peptide substrate for caspase 2 and 6 is detected. The assay is conducted in a 386-well plate format, and liquid chromatography-tandem mass spectrometry (LC-MS/MS) is employed to detect both the substrate peptide and the cleavage product for each caspase. This experimental design enables the high-throughput screening of inhibitors for caspases 2 and 6. The researchers utilized this approach to evaluate and optimize the structure–activity relationship (SAR) of various inhibitors for these two enzymes. One of the advantages of this method is its ability to avoid interferences in the detection of pharmacological activity caused by labeling the caspase substrates, which has been a drawback of fluorogenic assays that utilize rhodamine-labeled caspase substrates in particular [131,132]. An additional illustration of an MS-based technique for assessing caspase activity, particularly caspase 3, and identifying potential inhibitors involves the utilization of MALDI-TOF MS analysis to quantify the cleavage product of a peptide probe containing a caspase-3 cleavable segment (DEVD) linked with a quantifiable segment of FRGLRGFKC labeled by maleimide. Simultaneously, another peptide labeled with maleimide is analyzed as an internal standard, leading to the method being termed as “a dual maleimide labeling quantification”. This approach provides the benefits of rapid detection, consistent quantification, efficient high-throughput capability, and simplified sample preparation. The researchers showcased the method’s effectiveness in identifying caspase 3 activity in a cellular model of Parkinson’s disease [131,132].

##### N-Terminomics: Identifying Caspase Substrates

**Figure 6:**
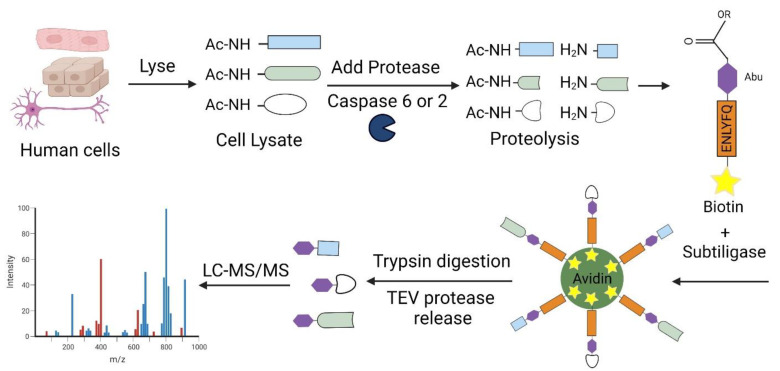
The schematic diagram outlines the principle behind one N-terminomics protocol, the Subtiligase approach, which was utilized to identify substrate proteins targeted by caspase-2 and caspase-6 directly. The process begins with the breakdown of human cells to isolate and collect cellular proteins, where most of them are naturally acetylated at their alpha-amines (AcNH-) unless they have undergone proteolysis during cell lysis. To prevent further proteolysis, endogenous proteases within the fresh cellular extract are effectively inhibited by adding protease inhibitors. Any excess thiol protease inhibitors are also neutralized with dithiothreitol (DTT) before introducing exogenous caspase. Subtiligase, an engineered peptide ligase, is then introduced along with a biotinylated ester, which facilitates the labeling of free N-termini of endogenous proteins and those newly generated through caspase cleavage. Avidin beads are utilized to capture the biotinylated proteins, which are subsequently digested with trypsin. The resulting N-terminal peptides, containing a non-natural Abu residue for identification, are released using TEV protease. Finally, liquid chromatography-tandem mass spectrometry (LC-MS/MS) is employed to identify and characterize the substrates and cleavage sites precisely. This innovative approach enables the direct elucidation of caspase-2 and caspase-6 substrate proteins and provides a robust method for unraveling the complexities of proteolytic events at the molecular level.

N-terminomics is a potent technique in proteomics and has played a pivotal role in uncovering and profiling caspase substrates (Figure 6) [133,134,135,136,137,138]. This approach has significantly advanced the understanding of protease substrates and their functions in diverse biological processes. The importance of N-terminomics in offering detailed information on cellular processes is evident through the continuous improvements in sample preparation, peptide enrichment, and bioinformatics tools [139,140]. The method focuses on profiling the N-termini of protein fragments through the use of advanced LC-MS/MS techniques and innovative methodologies like Two-Dimensional Differential In-Gel Electrophoresis (2D-DIGE), activity-based protein profiling (ABPP), Combined Fractional Diagonal Chromatography (COFRADIC), Terminal Amine Isotopic Labeling of Substrates (TAILS), Substrate-Targeted Ligase (Subtiligase), and Chemical enrichment of Protein Substrates (CHOPS). The growing use of N-terminomics in caspase research has enabled the development of the CaspSites database and web application, which offers investigators in the field a comprehensive resource and list of experimentally observed human caspase substrates [141]. The advantages of N-terminomics extend beyond human studies to include investigating other species. This is best exemplified in its application for investigating systems-wide endoproteolysis in the genome-reduced bacterial pathogen Mycoplasma hyopneumoniae. The method helped identify 669 N-terminal peptides from 164 proteins, with some of the proteins having multiple cleavage sites. The study helped lay out rules governing methionine excision, implicating several bacterial aminopeptidases [142]. In conclusion, N-terminomics is a potent and flexible technique that can significantly enhance the understanding of caspase substrates and their cellular functions, with implications that extend across a diverse range of organisms.

##### Unveiling Post-Translational Modifications

Caspase activity and function can be modulated by post-translational modifications (PTMs), of which phosphorylation has been the key regulator that can prevent caspase activation, promote disassembly of the large and small subunit of the enzyme, and suppress apoptosis [143]. MS is a powerful tool for identifying PTMs and can help uncover the complex regulation of these enzymes during the faithful execution of apoptosis [144]. In addition, the information harnessed from these studies can assist in developing therapeutic strategies for various disease states in which dysregulation in caspase activity has been involved. There are several examples in the literature on the use of mass spectrometry to help identify caspase phosphorylation sites. One example is the recent study by Lim et al., which identified in caspase 2 a highly conserved phosphorylation site at residue S384. The site has been deemed necessary for the enzyme’s catalytic activity and apoptosis initiation in response to mitotic insults. It is suggested as a biological marker that can help predict the sensitivity to apoptosis and treatment response in cancer [145].

##### Novel Inhibitors and Drug Discovery by MS

MS-based techniques have significantly contributed to developing caspase inhibitors, many of which hold therapeutic promise for diseases involving caspase dysregulation, such as cancer and neurodegeneration [146]. These techniques have been used to analyze the active site of caspases and identify key residues involved in substrate binding and cleavage, enabling the design and evaluation of specific inhibitors [51]. To this end, many of the common selective caspase inhibitors that have been designed based on substrate specificity profiles and are available through commercial vendors lack selectivity and show significant cross-reactivity towards other caspases [76]. The introduction of label-free LC/MS/MS-based enzymatic activity assays has been crucial in supporting the SAR studies for developing genuine caspase inhibitors of improved selectivity [131].

## 4. Conclusions: A Range of Tools for Revealing Caspase Activity

The repository of methods for investigating caspases witnessed remarkable expansion and evolution. These methods, which include classical antibody-based techniques and the more recently introduced methods based on cutting-edge state-of-the-art instrumentation, offer researchers significant leverage and flexibility in studying caspase dynamics in various biological contexts. While traditional methods have laid the foundation for the knowledge of the complex biological roles of caspases, the most recent advancements in detection methodologies have significantly expanded the capabilities, offering unprecedented insights into the intricate molecular details contributing to the functional roles of these enzymes in apoptosis. This comprehensive review has thoroughly examined the strengths and limitations of various methods, emphasizing their distinct capabilities in elucidating caspase activity within the cellular milieu (Table 1).

## Figures and Tables

**Table 1:** A comprehensive overview of techniques for detecting caspase activity in biological systems.

Technique	Description	Strengths and Weaknesses	Advancements
Techniques Based on Antibodies	Western blotting provides important information regarding protein levels; however, it may exhibit limitations in accurately quantifying activity.	Western blotting is a valuable technique for gaining insights into protein levels, although it does have limitations when it comes to accurately quantifying protein activity.	Current progress is centered on enhancing accuracy in quantifying activities in Western blotting, establishing standardized criteria for interpreting IHC results, and creating more user-friendly flow cytometry methods to increase accessibility.
	IHC enables visualization of caspase activation within tissues, providing crucial spatial context for understanding cellular processes.	IHC: Provides spatial context but subjective interpretation.	
	Flow cytometry detects caspase activity at the individual cell level, offering insights into cellular heterogeneity.	Flow cytometry: Reveals cellular heterogeneity but requires specialized equipment and expertise.	
Enzyme Assays	Fluorogenic substrates offer high sensitivity with minimal steps.	Fluorogenic substrates: High sensitivity but requires specific substrates.	Ongoing advancements include developing more versatile fluorogenic substrates to expand their applicability and exploring novel substrate designs for improved specificity and reliability in enzymatic assays.
	Luminescent assays provide enhanced sensitivity and reliability for high-throughput screening.	Luminescent assays: Reliable for screening but may lack specificity.	
	Fluorescence quenching assays detect caspase activity through substrate hydrolysis.	Quenching assays: Sensitive to hydrolysis but limited substrate options.	
	Caspase-specific inhibitors aid in identifying distinct caspase family members.	Caspase-specific inhibitors: Simplifies identification but may lack broad specificity.	
Advanced Detection Techniques	Label-free sensors eliminate background autofluorescence, enhancing sensitivity and accuracy.	Label-free sensors: Enhanced sensitivity but limited to specific detection types.	Advancements aim to broaden the applicability of label-free sensors, streamline SPR monitoring setups, develop less invasive in situ methods, simplify data analysis for real-time monitoring and HCS, and improve MS sample preparation protocols for enhanced caspase substrate profiling.
	SPR technology provides real-time monitoring of molecular interactions, providing a comprehensive understanding of molecular interactions.	SPR: Real-time monitoring but may require specialized equipment.	
	Various in situ assessment methods, such as in vivo imaging and single-molecule spectroscopy, enable a detailed examination and quantification of caspase activity within living organisms, thereby facilitating therapeutic monitoring.	In situ methods: Detailed quantification but may be invasive.	
	Real-time monitoring and HCS offer diverse approaches for evaluating cellular responses.	Real-time monitoring and HCS: Broad applications but complex data analysis.	
	MS methodologies advance caspase substrate identification and function understanding.	MS methodologies: Specific detection but may require extensive sample preparation and expensive instrumentation.	
